# A case of lethal spontaneous massive hemothorax in a patient with neurofibromatosis 1

**DOI:** 10.1186/s13019-014-0172-y

**Published:** 2014-10-29

**Authors:** Luisa Zacarias Föhrding, Timur Sellmann, Sebastian Angenendt, Detlef Kindgen-Milles, Stefan A Topp, Bernhard Korbmacher, Artur Lichtenberg, Wolfram T Knoefel

**Affiliations:** Department of General, Visceral and Paediatric Surgery, Heinrich Heine University Düsseldorf, Düsseldorf, Germany; Department of Anesthesiology, Heinrich Heine University Düsseldorf, Düsseldorf, Germany; Department of Cardiovascular Surgery, Heinrich Heine University Düsseldorf, Düsseldorf, Germany

**Keywords:** Neurofibromatosis type 1, Spontaneous hemothorax, Meningocele, Endovascular embolization

## Abstract

Neurofibromatosis type 1 is an autosomal dominant disease characterized by multiple dermatological disorders amongst others. Among the less frequent manifestations are vascular abnormalities. Here, we present a case of spontaneous massive hemothorax in a 39-year-old Caucasian woman with neurofibromatosis 1 and a thoracic meningocele with a lethal outcome despite extensive surgical intervention as well as intensive care measures. Spontaneous hemothorax is a rare, but potentially lethal complication of neurofibromatosis type 1, which necessitates quick and decisive intervention; endovascular embolization where possible, otherwise aggressive surgical intervention in unstable patients.

**Electronic supplementary material:**

The online version of this article (doi:10.1186/s13019-014-0172-y) contains supplementary material, which is available to authorized users.

## Background

Neurofibromatosis 1 (NF1) is an autosomal dominant disease characterized by café-au-lait macules, neurofibromas of any type, axillary and inguinal freckling and Lisch nodules in the iris. Additionally, numerous vascular manifestations have been reported [1]-[3]. Intrathoracic meningoceles have also been described in NF1 several times [4]-[6]. Spontaneous hemothorax is a rare but potentially lethal complication [7]-[9]. Here, we present a case of a lethal spontaneous hemothorax associated to an intrathoracic meningocele in a patient with NF 1.

## Case presentation

A 39-year old Caucasian female with known NF1 presented herself in the emergency department of an outside hospital with acute symptoms of thoracic pain and dyspnea. Her history was otherwise significant for an extensive thoracic myelomeningocele and status post-surgical correction of a thoracic scoliosis using a tibia bone graft 27 years earlier.

**Figure 1:**
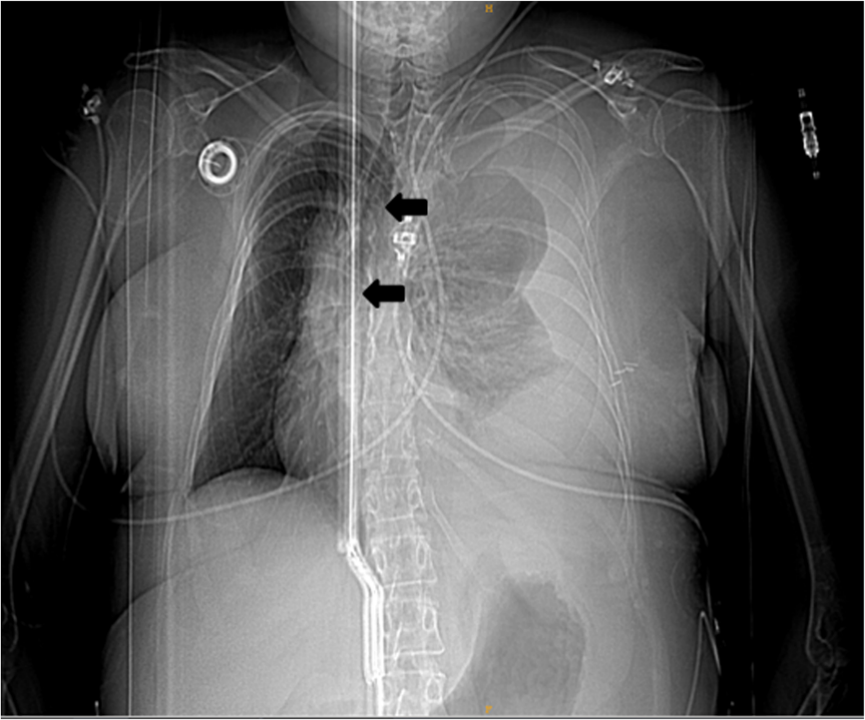
**Chest radiography showing extensive opacity in the left hemithorax.** Note the massive mediastinal shift (arrows).

**Figure 2:**
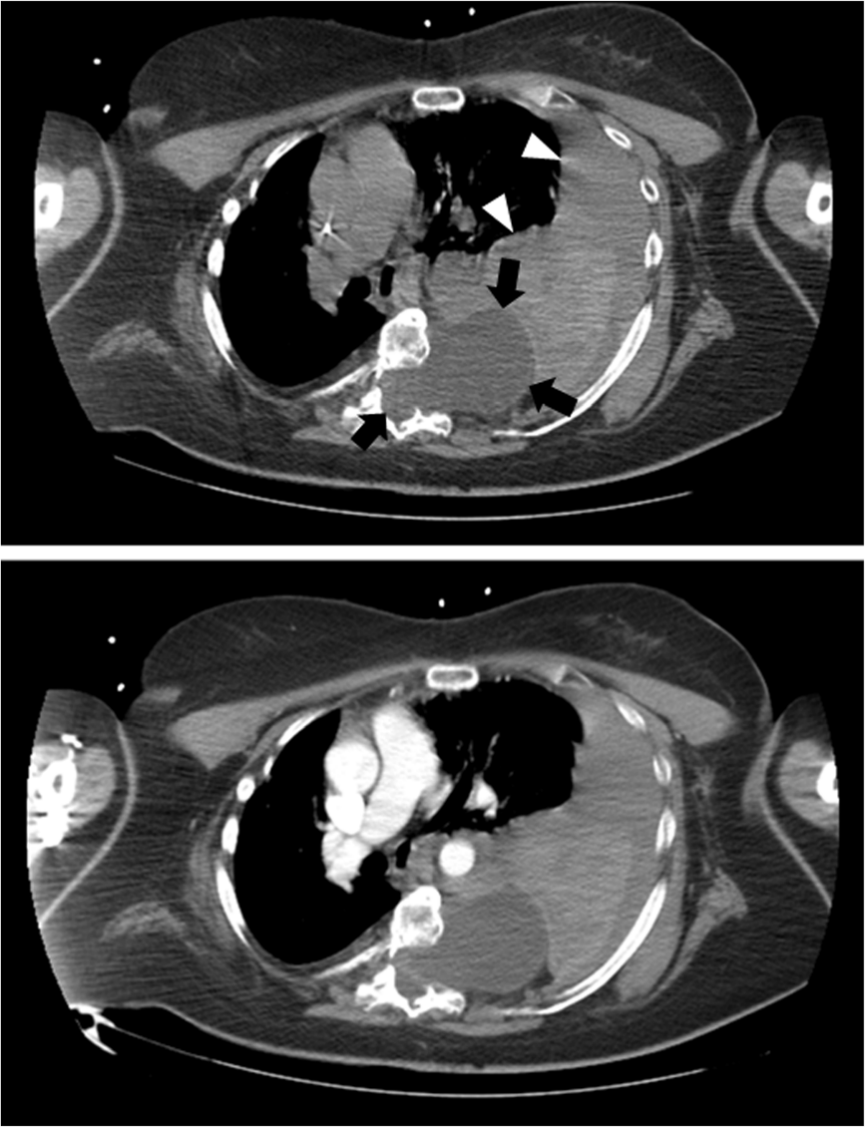
**Chest computer tomography (CT).** Upper figure: without contrast enhancement. Lower figure: with contrast enhancement. The arrows show the intrathoracic meningocele and the arrowheads show the hemothorax.

On physical examination at the outside hospital, she was awake, afebrile, blood pressure of 80/40 mmHg and a heart rate of 100 beats per minute. The initial haemoglobin was 12.5 g/dl, quickly deteriorating to 8.4 g/dl in the following hour. Chest x-ray (Figure 1) was performed 40 mins after admission and revealed an extensive mediastinal shift. Subsequent computer tomography (Figure 2) was performed 1h later at the outside hospital and revealed a massive hemothorax on the left side. The patient was intubated orotracheally because of hemorrhagic shock and imminent cardiopulmonary arrest and received seven packed red blood cell concentrates. A thoracic drainage was inserted prior to transfer. 4 h 10 min after admission to the outside hospital the patient was transferred to our institution and arrived there 1h later.

On admission, she was highly catecholamine-dependent. We found one left-thoracic suction drainage installed using Monaldi technique with 50 ml of blood in the drainage bottle. During placement of venous and arterial lines she suffered a cardiocirculatory arrest. Resuscitation was immediately initiated and we proceeded with performing an emergency exploratory left antero-lateral thoracotomy in the intensive care unit. Thereupon 2 liters of blood exsanguinated spontaneously. Despite massive blood transfusions (16 packed red blood cells, 2 thrombocyte concentrates, and 4 fresh frozen plasmas) and intravenous fluid administration no sufficient circulation could be established. Due to massive bleeding and very difficult operative conditions it was not possible to perform either an exploration of the thorax or an open cardiac massage via the same approach, which forced us to take a second approach through a median sternotomy for cardiac massage. Upon exploration of the thoracic cavity through the antero-lateral approach a large tumor, dorsal on the left side next to the vertebral column with massive bleeding, was detected. Despite several sutures and massive transfusions the bleeding could not be stopped and no sufficient blood pressure could be established. 7 h 30 mins after her first admission to the outside hospital and three hours after arriving at our institution the patient succumbed to the bleeding.

On autopsy multiple neurofibromas and several café-au-lait spots were scattered over the entire body surface. In the area of the tibia bone graft in the upper thoracic spine a protrusion of the dura mater of 8 × 10 cm was found. This meningocele showed a 1.8 cm perforation, which was probably due to the surgical procedures. Unfortunately, the autopsy could not reveal a distinct source of bleeding, but with a high probability, given the proximity to the oozing point, the bleeding must have originated from an intercostal artery.

## Conclusions

We hereby report a case of spontaneous hemothorax in a patient with neurofibromatosis 1 who went into cardiac arrest due to massive bleeding and finally died from uncontrollable hemorrhage. Despite immediate surgical intervention we were unable to locate the precise oozing point of the arterial bleeding due to the proximity to a large meningocele and the patient could not be resuscitated successfully.

So far, thoracic meningoceles have not been directly implicated in bleedings associated to NF1. The pathogenic relevance of the meningocele as the source of the bleeding, however, could not be supported by CT and autopsy. A deformity of the thorax and a mechanical stretching of an intercostal artery could have played a role [4]. However, the proximity of the oozing point to the large intrathoracic meningocele considerably complicated the operative procedure.

Since the case had an unfavorable outcome, one has to investigate whether the management was optimal. In NF1 surgical repair of bleeding vessels is complicated by arterial fragility due to intimal and medial dysplasia [10]. For this reason, endovascular embolization of arterial aneurysms is generally considered to be the treatment of choice in a stable hemodynamic situation as it is less invasive and more effective than surgical intervention [1],[9],[11],[12]. Video-assisted thoracoscopy (VATS) may be an alternative for evacuating a hematoma, especially after controlling the bleeding, e.g. by endovascular measures. During initial management with acute or even uncontrolled bleeding VATS seems to be suitable only in exceptional cases [13].

Since the patient in this case went directly into hemorrhagic shock and an unsatisfactory stabilization of the hemodynamic situation did not allow for an angiography, we considered an immediate thoracotomy as the only viable alternative. In line with this view, Miura *et al*. consider an emergent and aggressive surgical treatment to be indicated in patients in a hemodynamically unstable condition [14].

Collectively, it is conceivable that a faster transportation to our institution after a correct diagnosis of NF1-associated hemothorax and a timely embolization before the patient went into shock may have led to a favorable outcome. We therefore conclude that a quick diagnosis which facilitates early preparation of endovascular treatment is crucial in the management of spontaneous hemothorax in patients with NF1.

## Consent

Written informed consent was obtained from the patient’s next of kin for publication of this case report and any accompanying images. A copy of the written consent is available for review by the Editor-in-Chief of this journal.

## Authors’ original submitted files for images

Below are the links to the authors’ original submitted files for images. Authors’ original file for figure 1 Authors’ original file for figure 2

## Abbreviations

CT: Computer tomography
NF1: Neurofibromatosis 1
