# Supplementary figures and images for: A case of lethal spontaneous massive hemothorax in a patient with neurofibromatosis 1
Source: J Cardiothorac Surg. 2014 Oct 29;9:172. doi: 10.1186/s13019-014-0172-y (PMC4223165; doi:10.1186/s13019-014-0172-y)

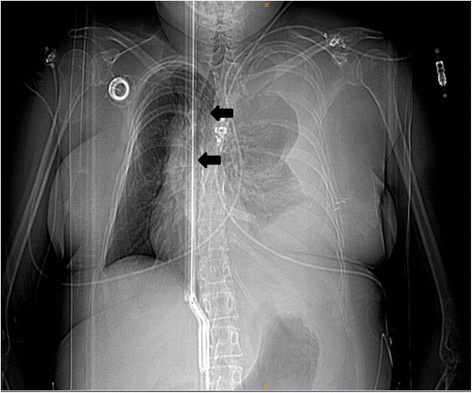

Supplement: Supplementary file 1 — Authors’ original file for figure 1 [file 13019_2014_172_MOESM1_ESM.gif]

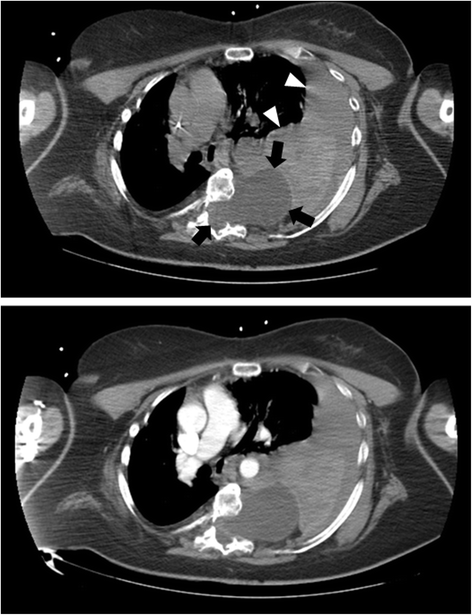

Supplement: Supplementary file 2 — Authors’ original file for figure 2 [file 13019_2014_172_MOESM2_ESM.gif]
